# Secretor Status Is Strongly Associated with Microbial Alterations Observed during Pregnancy

**DOI:** 10.1371/journal.pone.0134623

**Published:** 2015-07-31

**Authors:** Himanshu Kumar, Pirjo Wacklin, Massalin Nakphaichit, Eliisa Loyttyniemi, Somak Chowdhury, Yogesh Shouche, Jaana Mättö, Erika Isolauri, Seppo Salminen

**Affiliations:** 1 Funtional Foods Forum, University of Turku, Turku, Finland, 20520; 2 Finnish Red Cross Blood Service, Helsinki, Finland; 3 Department of Biotechnology, Faculty of Agro-Industry, Kasetsart University, Bangkok, Thailand, 10900; 4 Department of statistics, Faculty of Medicine, University of Turku, Turku, Finland, 20520; 5 Microbial Culture Collection, National Centre for Cell Science, Pune University Campus, Pune, Maharashtra, India, 411007; 6 Department of Paediatrics, University of Turku, Turku, Finland, 20520; Instutite of Agrochemistry and Food Technology, SPAIN

## Abstract

During pregnancy there are significant changes in gut microbiota composition and activity. The impact of secretor status as determined by genotyping *FUT2* (fucosyltransferase 2) gene was taken as one of the confounding factors associated with faecal microbiota changes during pregnancy. In this prospective study, we followed women during pregnancy (total = 123 of which secretors = 108, non-secretors = 15) and characterised their gut microbiota by quantitative polymerase chain reaction (qPCR), Fluorescence In situ Hybridisation (FISH), Denaturing Gradient Gel Electrophoresis (DGGE) and pyrosequencing. qPCR revealed that *C*. *coccoides group* counts decreased significantly in non-secretors in comparison to secretors (p = 0.02). Similar tendency was found by FISH analysis in *Clostridium histolyticum* and *Lactobacillus-Enterococcus* groups between the secretor and the non-secretor pregnant women. DGGE analysis showed significant decrease in richness of *Clostridium* sp. between secretor and non-secretor mothers during pregnancy. Pyrosequencing based analysis at phyla level showed that there is greater increase in Actinobacteria in secretors in comparison to non-secretors, whereas Proteobacteria showed more increase in non-secretors. Change in relative abundance of *Clostridiaceae* family from first to third trimester were significantly associated with secretor status of pregnant women (p = 0.05). Polyphasic approach for microbiota analysis points out that the host secretor status (FUT2 genotype) affects the gut microbiota during pregnancy. This may lead to altered infant gut microbiota colonization.

## Introduction

Microbiota colonising the intestinal tract generally maintains homeostasis and has a marked effect on human health. Pregnant women go through significant metabolic and physiological changes from the conception to the delivery and subsequent long term health impact are also observed [1, 2]. These metabolic changes often resemble obesity; like metabolic syndrome with increased blood glucose levels [3]. Developmental programming during pregnancy is affected by metabolic exposures induced by genetic variants, behavioural features and environmental factors [4]. We have earlier demonstrated that during pregnancy there is profound change in microbial composition from first trimester to third trimester, with an increase in Proteobacteria and Actinobacteria [5].

Association studies have reported that polymorphisms in gene like TLR-4 encoding innate mucosal immunity are associated with inter-individual differences observed in vaginal microbiota and pregnancy outcomes [6]. “Secretor status” (as determined by FUT2 genotype) was associated with incidence of urinary tract infection (UTI) in pregnant women [7]. Non-secretor pregnant women exhibited higher incidence of UTI infection than the secretors, potentially because of lack of ABO blood group antigens located on mucosa of urothelial cells [7]. FUT2 gene encodes for fucosyltransferase 2 enzyme, which synthesize the ABO antigens in secretions, such as intestinal mucosa. Non-secretors are homozygous for non-functional *FUT2*, single nucleotide polymorphism rs601338 (W143X, G428A). On an average 20% of individuals of European descent are non-secretors [8, 9]. In addition to UTI, recent reports also suggest that non-secretors are more prone to diseases like Crohn’s disease and type 1 diabetes [7, 10, 11]. Previously, our group has also shown that there are distinct differences in microbiota profile of adult secretors and non-secretors based on FUT2 genotype [9, 12]. Non-secretors were low in richness and abundance of bifidobacteria, *B*. *angulatum*, *B*. *catenulatum* when compared to secretors and several other genera such as *Clostridium* [9, 12].

In this study, we hypothesised that secretor status could be an important factor guiding the microbial changes observed during pregnancy.

## Material and Methods

### Sample collection and ethics statement

All subjects were selected from a prospective follow-up study of 256 pregnant women recruited for clinical study described elsewhere [5, 13]. Written informed consent was obtained from the women and the study protocol was approved by the Ethics Committee of the Hospital District of South-West Finland (registration number NCT00167700). The criteria for selection were availability of faecal samples at first trimester and third trimester, and whole blood samples. Altogether 123 women were selected for microbial and secretor analysis. EDTA anti-coagulated blood was kept at +4°C and subsequently stored at -80°C until DNA extraction. Stool samples were collected from the pregnant women at two time points during pregnancy in first trimester (T1) and in third trimester (T2). The stool samples were frozen at -18°C after collection at home and stored at -80°C until DNA extraction.

### DNA isolation and determination of secretor status

For DNA isolation, the stool samples were processed as described earlier [13]. Briefly, different aliquots were processed for DNA isolation, and fixing the cells for in situ hybridization analysis. For FISH analysis, the cells were fixed in 4% paraformaldehyde and stored in 50% ethanol at -20°C. From another aliquot, stool DNA was isolated using QIAmp DNA stool Mini kit (Qiagen, Hilden, Germany) [13]. Human DNA from blood samples was extracted using QIAamp DNA Blood Kit (Qiagen Inc, CA, USA) [9]. FUT2 genotype and secretor status were determined by genotyping the *FUT2* SNP rs601338 as described by Wacklin et al (2011, 2014).

### Quantitative PCR and FISH analysis

All the qPCRs were conducted according to the method described earlier [13]. PCR primers were used to target the *Bifidobacterium* genus, *B*. *longum*, *B*. *bifidum*, *B*. *adolescentis*, *B*. *catenulatum*, *Clostridium coccoides* group and *Clostridium leptum* group, *Bacteroides fragilis* group and *Akkermansia muciniphila* [13].PCR amplification and detection were performed on ABI 7300 system (Applied Biosystems, Foster City, CA). For FISH analysis the genus or group specific probes targeting eubacterial 16S rRNA gene, *Bacteroides-Prevotella* group, *Bifidobacterium* genus, *Clostridium histolyticum* group and *Lactobacillus-Enterococcus* group, have been adopted from the earlier reports [13, 14]. Flow cytometric analysis was performed with the use of a BD LSR II flow cytometer (Becton Dickinson and Co, Franklin Lakes, NJ) with 488-nm laser at 15mW. Data were analysed offline with the use of the BD FACSDIVA software (Becton Dickinson and Co, Franklin Lakes, NJ).

### PCR-DGGE and data analysis

Bacterial strains used as standard for DGGE analysis are described in (S1 Table) and strain specific culture conditions are described elsewhere [15]. PCR primers targeting *Bifidobacterium* spp., *B*. *fragilis* group, total bacteria, *C*. *coccoides- E*. *rectale* group have been adopted from an earlier report and are described by Endo et al. [15]. DGGE for all the bacterial groups was conducted with a DCode System (Bio-Rad Laboratories, CA). DGGE images were processed in Bionumerics software v 6.6 (Applied Maths, Belgium) for normalization and band detection [15]. Bands were matched using a tolerance of 1% and relative abundance of each species in each bacterial group was determined according to the BioNumerics software. DNA bands were matched by migration comparison with DNA bands of respective reference strain.

### Pyrosequencing analysis

All the pyrosequencing based sequencing data was generated during our earlier collaborative study and OTU data was adopted from this study [5]. Relative abundance of phyla and family at first and third trimester of pregnancy were compared between secretors and non-secretors taking time as factor for interaction.

### Statistical analysis

FISH and qPCR analysis for bacterial counts were compared at first trimester between secretor status with one-way analysis of variance. Trimesters 1 and 3 were handled as repeated measures (within-factor) and secretor status/genotype as (between) factor. Interaction was introduced to test if the microbial changes observed from first trimester to third trimester are associated with secretor status. Microbial changes were based on bacterial counts from qPCR & FISH analyses, and relative abundance from pyrosequencing, respectively. Analyses were performed with hierarchical linear mixed models (SAS PROC MIXED). Normal distribution assumption was examined from studentized residuals. Additionally, for non-normal qPCR counts, Kruskal-Wallis test was used to test the difference between secretor statuses. Analyses were performed with SAS System, version 9.3 for Windows.

For DGGE and Pyrosequencing data, constrained clustering method, redundancy analysis (RDA), was applied to analyze differences in bacterial composition between non-secretors and secretors in first and third trimester. RDAs based on relative abundance in the phyla or family level were assessed using R, version 3.0.2 [16]and its extension package vegan [17]. The data was Hellinger transformed for RDA. ANOVA for RDA clustering was performed with a full model using 999 permutations in R as described in Wacklin et al. 2011 and 2014.

Richness and Shannon diversity indices were calculated based on matrices exported from Bionumerics and imported to Microsoft Excel. Shannon diversity index, H’, was calculated using the equation H’ = -Σ Pi ln (Pi). Pi is the importance probability of the bands in a gel lane and is calculated as Pi = ni/N where ni is the

intensity of band and N is the sum of intensities of all bands in the densitometric profile. Man Whitney test was used to compare the difference in richness and diversity between secretor status at two time points (T1 & T2). The statistical analysis was performed with IBM SPSS Statistics for Windows (Version 21.0. Armonk, NY: IBM Corp.)

## Results

### Secretor Status

Sequencing of secretor genotype or FUT2 genotyping (single nucleotide polymorphism, rs601338) was carried out from the blood samples of 123 women. Among them, 108 women were secretor positive (42 women with genotype GG, 66 women with genotype GA), and 15 women were found to be non-secretors (with genotype AA). qPCR, FISH and DGGE analysis was carried out on a set of 71 stool samples (62 secretor and 9 non-secretor), and second set of 52 stool samples (46 secretor and 6 non-secretor) were analysed by pyrosequencing as described in our previous study [5].

### Microbial composition by qPCR

Significant differences were observed in specific microbial composition of secretor (n = 62) and non-secretor (n = 9) pregnant women by qPCR analysis. Importantly, there was a significant decrease (p = 0.02) in the mean change of *C*. *coccoides* group counts (from T1 to T2) in non-secretors than in secretors (Table 1). Additionally, bifidobacteria counts were found to be significantly different at first trimester (Baseline) (p = 0.04) between secretor and non-secretors (i.e. lower counts in non-secretors, Table 1), which could also be attributed to the significant difference observed later during pregnancy.

**Table 1:** Quantitative PCR analysis for bacterial counts in faecal samples of pregnant women at first trimester and third trimester.

	Secretor (n = 62)				Non-secretor (n = 9)						
	First trimester		Third trimester		First trimester		Third trimester				
	Mean log cells/g	(S.D)	Mean log cells/g	(S.D)	Mean log cells/g	(S.D)	Mean log cells/g	(S.D)	P value^a^	P value^b^	P value^c^
Bifidobacterium genus^d^	11.52	0.28	10.79	0.62	11.28	0.56	10.44	0.66	0.04	0.04	0.40
*C*. *coccoides* group^d^	10.53	0.43	8.95	0.67	10.66	0.38	8.40	0.33	0.29	0.11	0.02
*C*. *leptum* sub-group^d^	9.80	0.43	9.09	0.78	9.71	0.28	8.69	0.41	0.14	0.28	0.11
*Bacteroides fragilis* group^d^	6.13	0.91	5.72	1.30	6.44	0.75	5.49	0.59	0.84	0.93	0.13

a = Baseline differences at first trimester

b = test for secretor status as fixed effect

c = interaction between secretor and time as factors

d = P for time effect <0.002, SD = Standard deviation

### Microbial composition by FISH analysis

FISH analysis demonstrated significant differences in microbiota composition between the secretor and the non-secretor pregnant women. Significant decrease (p = 0.04) in counts of *Bacteroidetes-Prevotella* group was observed from T1 to T2 in non-secretors in comparison to secretors (Table 2). Counts of *Clostridium histolyticum* group and *Lactobacillus-Enterococcus* group exhibited trend towards significance when mean count change was compared from T1 to T2 between secretors and non-secretors (Table 2). Additionally at baseline (T1), significance difference was observed when *Clostridium histolyticum* group counts between the secretors and the non-secretors (Table 2). However, the mean change in counts of total bacteria and *Akkermansia muciniphila* group were not associated with secretor status (Table 2).

**Table 2:** Fluorescent in situ hybridization analysis for bacterial counts in faecal samples of pregnant women at first trimester and third trimester.

	Secretor (n = 62)				Non-secretor (n = 9)						
	First trimester		Third trimester		First trimester		Third trimester				
	Mean log cells/g	(S.D)	Mean log cells/g	(S.D)	Mean log cells/g	(S.D)	Mean log cells/g	(S.D)	P value^a^	P value^b^	P value^c^
Total bacteria^d^	9.19	0.28	9.09	0.33	9.15	0.31	8.85	0.32	0.04	0.12	0.14
*Bifidobacterium* genus^d^	8.25	0.3	8.09	0.4	8.27	0.45	7.96	0.55	0.48	0.45	0.36
*Bacteroides*-*Prevotella* group^d^	8.03	0.28	7.84	0.31	8.05	0.33	7.56	0.32	0.07	0.1	0.04
*Clostridium histolyticum* group^d^	8.02	0.28	7.89	0.35	7.99	0.34	7.6	0.28	0.03	0.07	0.08
*Lactobacillus*-*Enterococcus* group^d^	8.08	0.26	7.9	0.33	8.1	0.27	7.69	0.29	0.18	0.24	0.09
*Akkermansia muciniphila* ^d^	7.94	0.28	7.73	0.33	7.94	0.33	7.48	0.28	0.08	0.11	0.1

a = Baseline differences at first trimester

b = test for secretor status as fixed effect

c = interaction between secretor and time as factors

d = P for time effect <0.0007, SD = Standard deviation

### Microbial composition by DGGE analysis

At first trimester the diversity of dominant bacteria was similar between the secretors and the non-secretors. However, during the pregnancy at third trimester the diversity and richness was reduced in the non-secretors in comparison to the secretors (S1 Fig). *C*. *coccoides-Ruminococcus* group showed trend towards significance in richness at first trimester (p = 0.07), and found to be significantly different at third trimester when compared between the secretor and the non-secretor pregnant women (p = 0.033). Additionally, diversity analysis of *C*. *coccoides* group exhibited significant difference at third trimester of pregnancy (p = 0.04) (S1 Fig).

**Fig 1:**
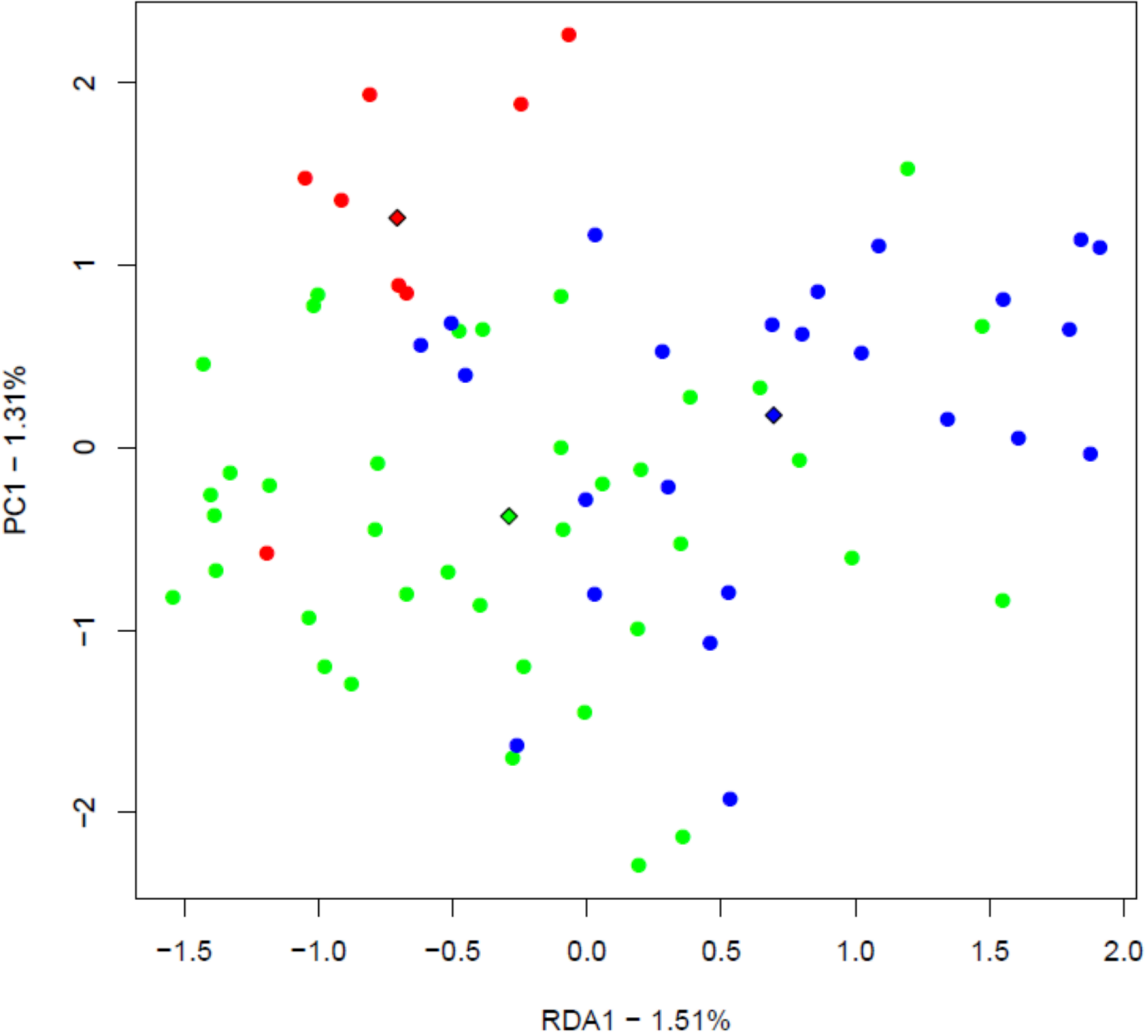
RDA plot for microbiota analysis by DGGE- *C*. *coccoides* group at First trimester based on genotype (p = 0.005) (non-secretors genotype (AA) in red, secretor genotype (GG) in blue and secretor genotype (AG) in green). Triangles indicate centroids of study groups.

RDA analysis of PCR-DGGE revealed that the composition of *C*. *coccoides* group was found to be significantly different at first trimester of pregnancy in the women with different genotypes (Fig 1). The composition of other examined groups (total bacteria and bifidobacteria) were not significantly differing between women categorised based on secretor status or genotypes in either time points (data not shown).

### Microbial composition by pyrosequencing analysis

**Fig 2:**
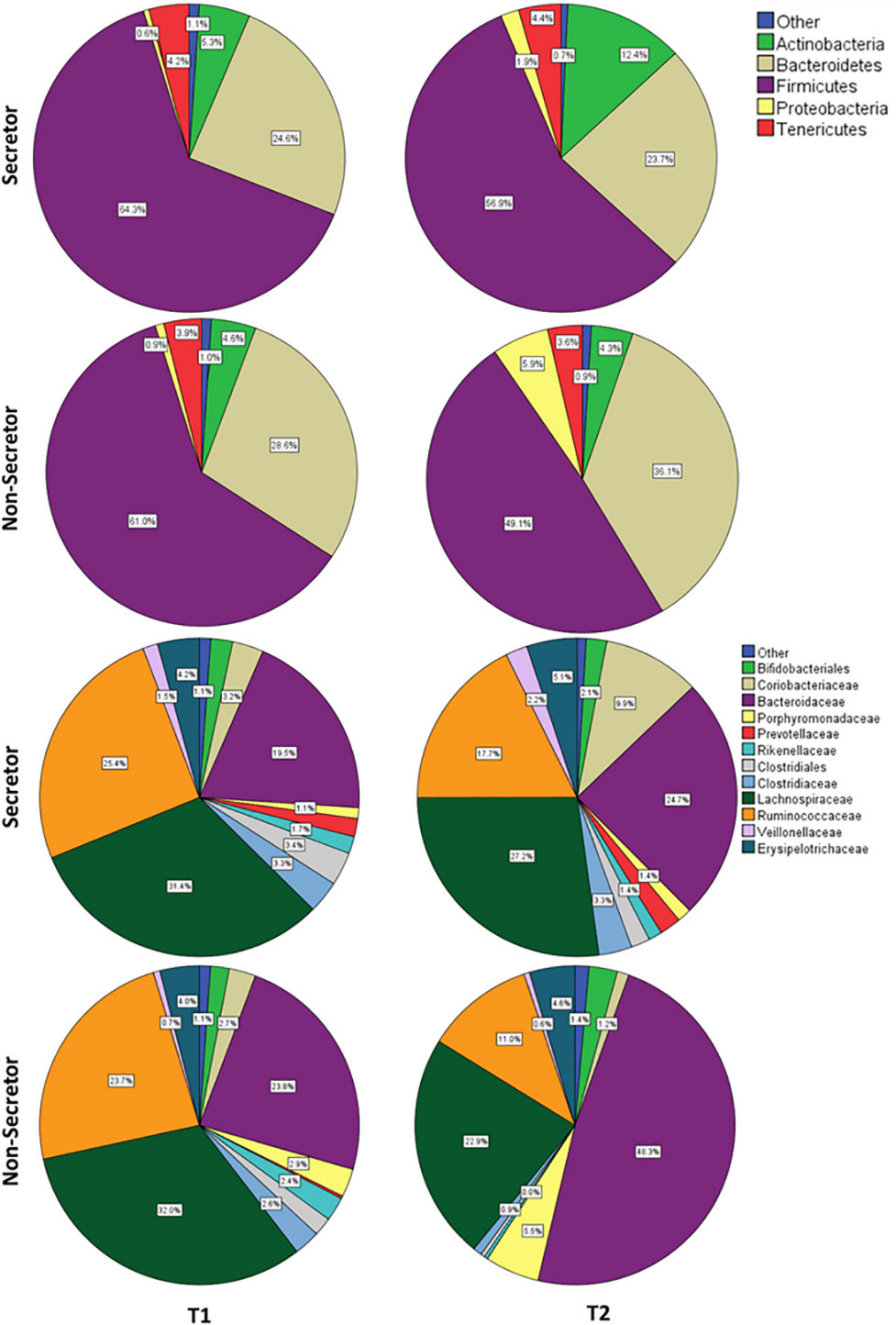
Pie chart of percent mean relative abundance of microbial composition as determined by pyrosequencing A) Phylum B) Family level (with percent relative abundance of more than 1%), at First trimester (T1) and at Second trimester (T2) compared between Secretors and Non-secretors.

**Fig 3:**
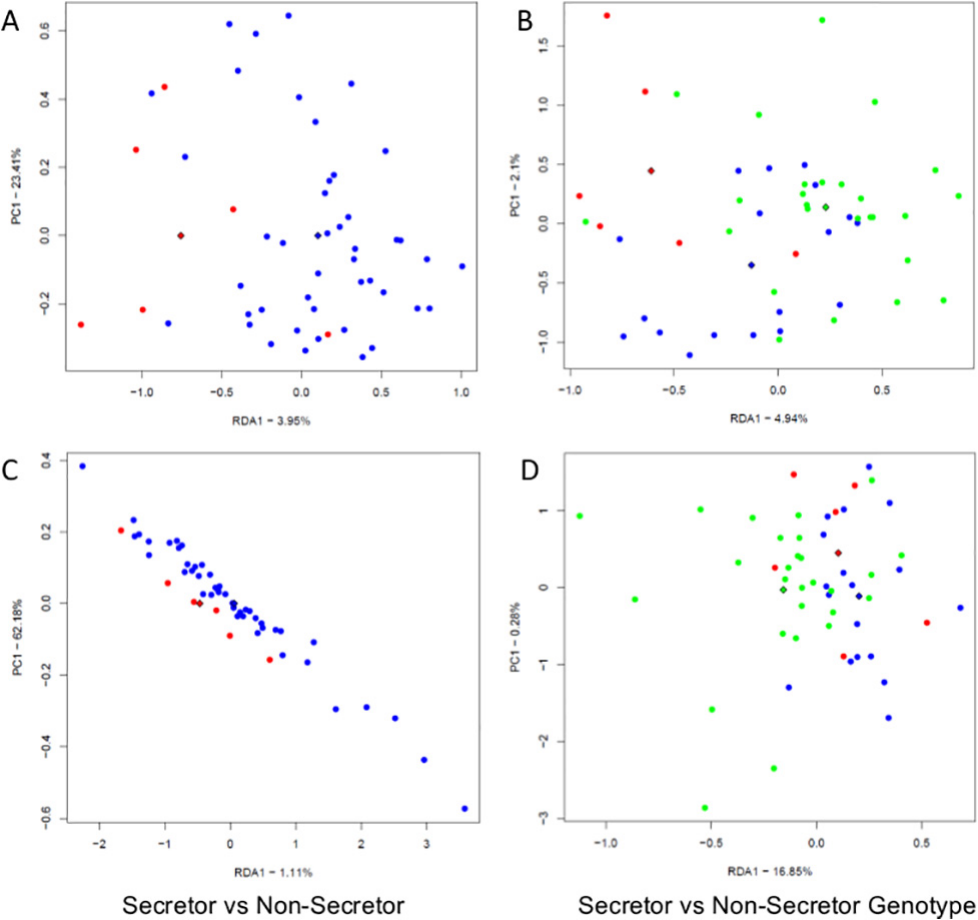
RDA plots for pyrosequencing based microbiota analysis: Family level (A&B) microbial composition was significantly different at third trimester when compared A) Secretors (blue circles) vs Non Secretors (red circles) (p = 0.04); B) and genotypes (AA = red, GA = green, GG = blue) (p = 0.01). **Phyla level** (C&D) difference was found to be different at first trimester when C) Secretor status D) Genotypes are compared (p = 0.005). Triangles indicate centroids of study groups.

At phyla level, pyrosequencing data analysis revealed that there is an increase in Actinobacteria in secretor women, while in non-secretors there is a higher increase in the relative abundance of Proteobacteria. Interestingly, change in relative abundance of *Clostridiaceae* family was found to have significant decrease in non-secretors in comparison to secretors (p = 0.05) (Fig 2A and 2B). RDA analysis for compositional differences of bacterial communities at phyla level revealed that bacterial composition significantly differed (p = 0.005) when FUT2 genotypes were compared at first trimester (Fig 3) while this trend was not observed at third trimester. However, at third trimester secretors and non-secretors formed different clusters (p = 0.04) in the RDA analysis for bacterial composition at the family level and differences were also significant when FUT2 genotypes were compared (p = 0.01) indicating that the microbial compositional difference based on genotypes (Fig 3).

## Discussion

Earlier studies have reported that gut microbiota is modified during pregnancy and by the third trimester there is significant increase in the relative abundance in Proteobacteria and Actinobacteria [5]. Interestingly, this increase was not related to any of the measured health parameters or diet. In another study, we have reported that BMI could be an additional factor contributing to the microbial changes observed during pregnancy, such as an increase of *Bacteroides* group and *Staphylococcus aureus* was more pronounced in obese women in comparison to normal weight women [13]. However, levels of bifidobacteria were higher in normal weight women suggesting normalization of microbiota associated inflammation observed during pregnancy.

Recently, it has been reported that host genetics play a significant role in shaping the composition of gut microbiome [9, 10, 12], which could in turn impact the host metabolism [18]. In this study, we report that secretor status could be one of the genetic contributors for the changes observed during pregnancy, as the secretors showed higher levels of Actinobacteria at third trimester in comparison to non-secretor women. qPCR analysis of bifidobacteria also revealed that there are significant differences in the colonisation among secretor and non-secretors. In adults, the numbers of bifidobacteria were also reported to be higher is secretors than non-secretors [12]. This could be due to specific changes which were observed in *B*. *adolescentis* among secretor and non-secretors. Bifidobacteria are known to be associated with healthy intestinal microbiota and considered safe for probiotic applications. Reduced numbers of bifidobacteria in non-secretors have been associated with an increased susceptibility to intestinal disorders and higher levels in secretors may be attributed to the normalization of inflammatory status observed during pregnancy [2].

In non-secretors the change in the relative abundance of Proteobacteria between first and third trimester was prominent. Proteobacteria are also implicated to have a role in inflammation associated microbiota deviations reported in subjects with Inflammatory Bowel Disease [19]. Which could explain the higher susceptibility of non-secretors are also known to have higher susceptibility to intestinal related disorders like coeliac disease [20] and Crohn’s disease [10]. Our study may help to explain this observation that non-secretor status may drive higher change in Proteobacteria in comparison to secretor women during pregnancy.

In a previous study, DGGE analysis revealed that *Clostridium* cluster IV and *Clostridium* cluster XIV showed a trend for differences between secretor and non-secretor adults [9]. In DGGE, we found clustering based on *Clostridium coccoides* group and FUT2 genotype. Interestingly, *Clostridiaceae* family was also found to significantly different between secretors and non-secretors, with higher decrease in non-secretors. Clostridium spp. has been reported for its role in maintenance of immunity [21]. High number of *Clostridia* sp., *Bifidobacterium* sp. and *Bacteroides* group in secretors suggest potential glycan utilizing capacity of these microbes [22–24]. Moreover, absence of fucosylation in mucus layer may be associated with alter intestinal permeability potentially increasing the susceptibility to intestinal inflammation.

Utilising multiple methods for microbiota analysis, we could assess the comprehensive insight into the predominant bacterial diversity by pyrosequencing and DGGE, bacterial group specific assessment by FISH and accurate quantification by qPCR. Specific microbial variations in significance observed in microbiota analysis by different methods could be ascribed to technical differences due to primers and/or analysis method, or due to low number of non-secretors in statistical analysis. However, there are specific microbial changes such as Clostridia genera, which are associated with secretor status of pregnant women.

This study highlights the effect of host genotype on microbial alteration even at the critical period of pregnancy. We used polyphasic approach to understand the effect of FUT2 genotype on gut microbiota changes during pregnancy. Our analysis further suggests that pregnant women have altered microbiota compared to non-pregnant adults and FUT2 or secretor status could acts as one of the contributing factors for microbiota changes observed during pregnancy. As such differences may lead to altered microbiota transfer from mother to the infant, this phenomenon deserves further assessment.

## Supporting Information

S1 Fig DGGE analysis for microbial richness and diversity of Total bacteria and *Clostridium coccoides* group, (1 = secretor, 2 = non-secretor, at T1 (first trimester) and T2 (third trimester)). (TIF)

S1 Table DGGE standard strains for specific bacterial groups. (DOCX)

S2 Table Quantitative PCR analysis for Bifidobacteria group and *Akkermansia muciniphila* in faecal samples of pregnant women at first trimester and third trimester with secretor and non-secretor as the factors. (DOCX)
