# Supplementary figures and images for: Secretor Status Is Strongly Associated with Microbial Alterations Observed during Pregnancy
Source: PLoS One. 2015 Jul 31;10(7):e0134623. doi: 10.1371/journal.pone.0134623 (PMC4521695; doi:10.1371/journal.pone.0134623)

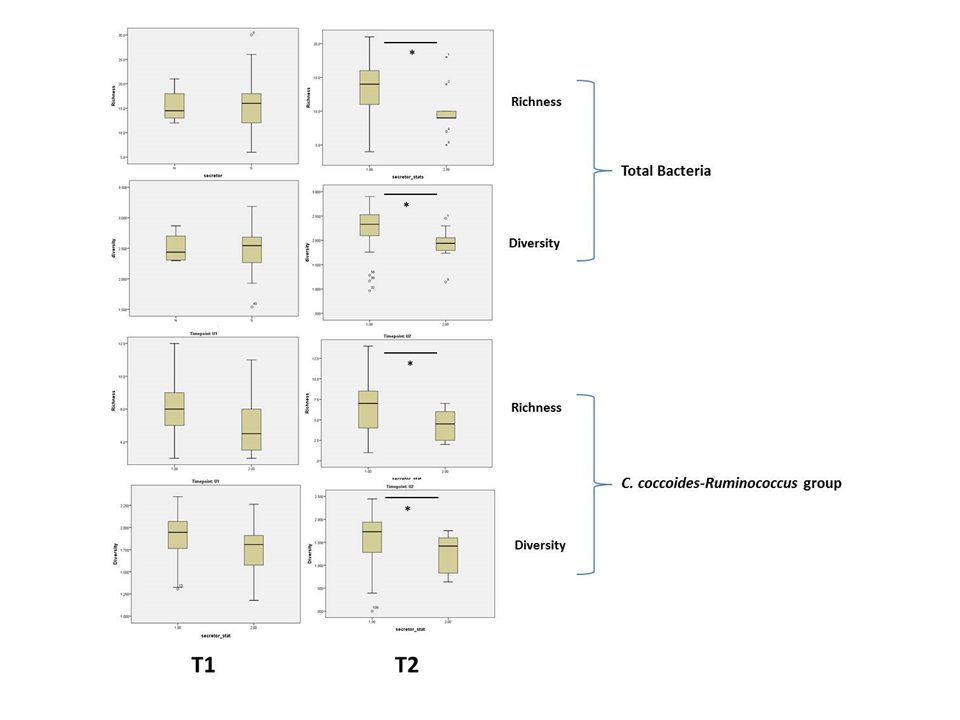

Supplement: S1 Fig — (TIF) [file pone.0134623.s001.tif]
