# Supplementary material for: Secretor Status Is Strongly Associated with Microbial Alterations Observed during Pregnancy
Source: PLoS One. 2015 Jul 31;10(7):e0134623. doi: 10.1371/journal.pone.0134623 (PMC4521695; doi:10.1371/journal.pone.0134623)
Supplement: S1 Table — (DOCX) [file pone.0134623.s002.docx]

**S1 Table**. **DGGE standard strains for specific bacterial groups**

1. Total bacteria
2. *Bacteroides fragilis* DSM 2151
3. *Lactobacillus gasseri* DSM 20243
4. *Leuconostoc mesenteroides subsp. mesenteroides* DSM 20343
5. *Lactobacillus salivarius* DSM 20555
6. *Bifidobacterium breve* DSM 20213
7. *Bifidobacterium longum* DSM 20219
8. *Bifidobacterium bifidum* DSM 20456
9. *Bifidobacterium angulatum* JCM 7096
10. For Bifidobacteria group
11. *B. adolescentis* DSM 20083
12. *B. bifidum* DSM 20456
13. *B. angulatum* JCM 7096
14. *B. longum* DSM 20219
15. *B. breve* DSM 20313
16. *B. catenulatum* DSM 16992
17. *C. coccoides-Eubacterium-Ruminococcus* group
18. *C. nexile* DSM 1787
19. *Blautia coccoides* DSM 935
20. *Eubacterium halii* DSM 3353
21. *Blautia glucerasea* DSM 22028
22. *Ruminococcus obeum* DSM 23538
23. *Ruminococcus gnavus* CCUG 33437
24. *B. fragilis* group
25. *B. vulgatus* DSM 1447
26. *B. dorei* DSM 17855
27. *B. massiliensis* DSM 17679
28. *B. ovatus* DSM 1896
29. *B. fragilis* DSM 2151
30. *B. caccae* CCUG 38735
31. *B. coprocola* CCUG 54635
32. *B. uniformis* DSM 6597
33. *Parabacteroides distasonis* CCUG 4941
