# Supplementary material for: Secretor Status Is Strongly Associated with Microbial Alterations Observed during Pregnancy
Source: PLoS One. 2015 Jul 31;10(7):e0134623. doi: 10.1371/journal.pone.0134623 (PMC4521695; doi:10.1371/journal.pone.0134623)
Supplement: S2 Table — (DOCX) [file pone.0134623.s003.docx]

**S2 Table**. **Quantitative PCR analysis for Bifidobacteria group and *Akkermansia muciniphila*** in faecal samples of pregnant women at first trimester and third trimester with secretor and non-secretor as the factors

|  | **Secretor (n=62)** | | | | **Non-Secretor (n=9)** | | | |  |  |
| --- | --- | --- | --- | --- | --- | --- | --- | --- | --- | --- |
|  | First trimester | | Third trimester | | First trimester | | Third trimester | |  |  |
|  | Median | (Q1,Q3) | Median | (Q1,Q3) | Median | (Q1,Q3) | Median | (Q1,Q3) | P value^a^ | P value^b^ |
| *B. longum* | 11.10 | (10.87,11.34) | 10.19 | (9.67,10.41) | 11.06 | (10.31,11.25) | 9.55 | (9.19,10.33) | 0.75 | 0.91 |
|  |  |  |  |  |  |  |  |  |  |  |
| *B. bifidum* | 6.78 | (5.36,10.54) | 7.01 | (5.36,8.82) | 5.36 | (5.36,9.95) | 5.36 | (5.36,8.27) | 0.81 | 0.38 |
|  |  |  |  |  |  |  |  |  |  |  |
| *B. adolescentis* | 9.51 | (5.90,10.37) | 7.19 | (5.68,9.98) | 9.61 | (5.96,10.54) | 8.54 | (6.30,9.91) | 0.72 | 0.04 |
|  |  |  |  |  |  |  |  |  |  |  |
| *B. catenulatum* | 6.47 | (5.14,10.18) | 6.80 | (5.14,8.87) | 7.31 | (5.71,10.23) | 7.26 | (5.14,8.72) | 0.32 | 0.60 |
|  |  |  |  |  |  |  |  |  |  |  |
| *Akkermansia muciniphila* | 8.00 | (6.19,9.06) | 7.88 | (5.76,8.88) | 9.07 | (8.32,9.12) | 8.41 | (7.41,8.76) | 0.40 | 0.19 |

a=change between first and third trimester compared between secretor vs non-secretor performed with Wilcoxon rank sum test, b= change between first and third trimester change compared between genotypes [secretor (GG, GA), non-secretor (AA)] performed with Kruskal-Wallis test
